# Intramuscular Short-term ACTH Test for the Determination of Adrenal Function in Children: Safe, Effective and Reliable

**DOI:** 10.4274/jcrpe.galenos.2019.2019.0099

**Published:** 2020-09-02

**Authors:** Elif Özsu, Zeynep Şıklar, Esra Bilici, Ayşegül Ceran, Rukiye Uyanık, Tuğba Çetin, Zehra Aycan, Merih Berberoğlu

**Affiliations:** 1 Ankara University Faculty of Medicine, Department of Pediatric Endocrinology, Ankara, Turkey

**Keywords:** Adrenal insufficiency, intramuscular ACTH, childhood, reliability

## Abstract

**Objective:**

Standard short adrenocorticotropic hormone (ACTH) stimulation test (SST) has traditionally been used for assessing adrenal gland fuction by intravenous (iv) application. However the iv form is not readily available in all countries, including Turkey. The aim of this study was to evaluate the effectiveness of the intramuscular (im) SST.

**Methods:**

Patients underwent im SST with suspected adrenal insufficiency (AI) and hyperandrogenism. The SSTs were done with 250 mcg ACTH (Synacthen Depot ampul, concentration 1 mg/mL). The cases were divided into two groups: suspected AI (group 1 n=87); and hyperandrogenism group (group 2 n=124). Definite AI was defined as peak cortisol <18 μg/dL, suspected AI as a peak cortisol of 18-21 μg/dL and normal result was defined as a peak cortisol ≥22 μg/dL.

**Results:**

The mean age of the patients was 11.7±5.2 years. In 164 patients (78%) all of the peak cortisol tests were normal (≥22 mcg/dL). The rates were 64% and 88% in group 1 and 2, respectively. Only 8.5% (n=18) of all cases had an inadequate peak cortisol response of <18 mcg/dL. On follow up, 15 patients whose peak cortisol was <18 mcg/dL needed cortisol therapy. Of all cases 3.3% (n=8) had 17-OHP ≥10 ng/dL. Clinical findings suggestive of non-classical congenital adrenal hyperplasia and/or mutation were found in six of these cases (75%). No local and systemic side effects or allergic reactions were observed in any patient.

**Conclusion:**

IM ACTH SST is a safe, effective and reliable test in children with suspected AI. There were no local or systemic side effects, supporting the reliability of the im ACTH test.

**What is already known on this topic?**

Intravenous (iv) short synacthen test has commonly been used to detect adrenal insufficiency (AI) in childhood. However, the iv form of adrenocorticotropic hormone (ACTH) is not readily available in all countries.

**What this study adds?**

The intramuscular (im) form of ACTH test can be used as an alternative to iv form to detect cases with AI. The im form of ACTH test is safe and reliable test for suspected AI in childhood.

## Introduction

The clinical presentation of adrenal insufficiency (AI) is very variable. Severe overt adrenal failure is usually relatively easy to diagnose; however milder AI may be much more clinically challenging. Thus dynamic tests are extremely important in detecting subtle (intermediate) AI. It has been recommended to perform dynamic test in every suspected case (1). Although the insulin tolerance test (ITT) has been used as a gold standard test for the diagnosis of AI, it is used with caution because of the risk of seizures in patients with epilepsy and possible morbidity, especially in infants and in patients with cardiovascular diseases (1,2).

The ITT may be replaced by a short adrenocorticotropin hormone (ACTH) stimulation test (SST), due to a better safety profile. However the ACTH preparations used for an intravenous (iv) SST test are not readily available in Turkey and require an official application for prescribing of overseas medicine. Due to this limitation, use of the intramuscular (im) form, which is easily accessible and cheaper, has been used to replace the iv preparation in order to evaluate the adrenal axis of patients without overt AI. The easily available im-ACTH preparations in Turkey include the original molecule, tetracosactide, known as Synacthen**^®^** Depot, which contains 50 units/mL of ACTH (Maalincrodot Speciality Pharmaceuticals Ltd, Ireland Limited College Business & Technology park, Curiserath, Blanchardstown, Dublin 15, Ireland).

The aim of this study was to evaluate the effectiveness of the SST using this im preparation and to correlate the findings with the clinical and acquired peak responses and basal cortisol results.

## Methods

The characteristics and data of the patients including age, gender, complaints, laboratory results and other demographics, undergoing im-ACTH test between 2010 and 2018 were extracted from the hospital records system and retrospectively analyzed. The inclusion criteria were patients aged <18 years who were tested because of suspected AI and all patients who were admitted with hyperandrogenism and underwent SST to investigate the possibility of non-classical congenital adrenal hyperplasia (NCCAH).

**Figure 1:**
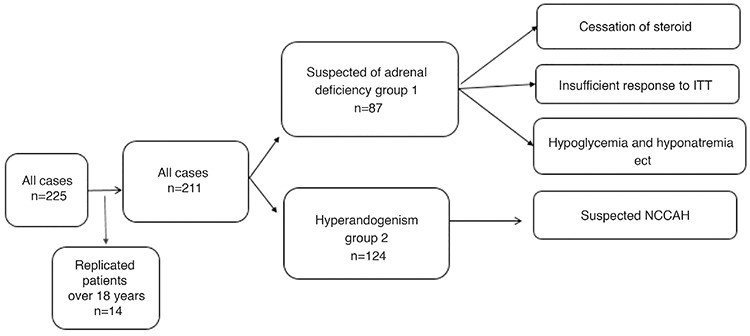
Classification of selected cases according to etiological factors ITT: insuline tolerance test, NCCAH: non-classical congenital hyperplasia

The cases were divided into two groups: suspected AI (group 1); and hyperandrogenism group (group 2). Group 1 consisted of cases that had AI with low basal cortisol and poor cortisol response to ITT. Group 2 had presented with findings of virilization, such as premature adrenarche, hirsutism and a clinical suspicion of NCCAH (see Figure 1).

The SSTs were performed with 250 mcg ACTH (Synacthen Depot ampoule, concentration 1 mg/mL) in the patients over two years of age. Blood samples were taken for measurement of cortisol, dehydroepiandrosterone sulfate (DHEAS) and 17-hydroxyprogesterone (17-OHP) at 0, 30 and 60 minutes after administration of the im-ACTH. Cortisol and DHEAS concentrations were analysed on a Beckman Coulter analyser (Beckman Coulter 250 S. Kraemer Blvd, Brea 92821, USA) by immunoassay method and 17-OHP concentration was measured by using a radioimmunoassay (DIA Source Immunoassay SA, Belgium). AI was defined as peak cortisol <18 µg/dL, suspected AI as a peak cortisol of 18-21 µg/dL and a normal result was defined as a peak cortisol ≥22 µg/dL. Genetic analysis for 21 hydroxylase gene mutations were performed in patients with a peak 17-OHP >10 ng/dL. Sensivity, specificity, positive predictive value (PPV) and negative predictive value (NPV) were calculated. In patients with a definitive diagnosis indicated by the results of the ACTH SST, findings during clinical follow-up were also investigated.

### Statistical Analysis

Statistical Package for Social Sciences version 22.0 (IBM Inc., Chicago, IL, USA) was used for all statistical analyses. Descriptive statistical analysis of the data was performed. Data distribution was assessed for normality using the Shapiro-Wilk test. Data distribution was non-parametric so groups were compared with Mann-Whitney U test. A p<0.05 was considered significant.

The study was approved by the Ethics Committee of Ankara University, with the decision number: 02-139-19.

## Results

Over the study period 225 patients who had undergone SST were evaluated. Fourteen patients were excluded because of duplicated patients or being aged over 18 years old. Thus the final number of study patients was 211. The mean age of the patients was 11.7±5.2 years and 69.2% were female. Of the 211 patients, 87 had been assessed for suspected AI (group 1) while 124 patients had been assessed for hyperandrogenism (group 2). In 164 patients (78%) all of the peak cortisol tests were normal that is ≥22 mcg/dL (equivalent to ≥600 nmol/L). This proportion was 64% and 88% in group 1 and 2 respectively. In 29 (13.7%) patients the peak cortisol was 18-21 µg/dL (equivalent to 500-599.9 nmol/L). Only 8.5% (n=18) of all cases had had an inadequate peak cortisol response of <18 µg/dL (equivalent to <500 nmol/L). Age of 11 patients were under 2 years. Their test endications were (one patient can have 2 indications for test) 5% of all patients. The distrubution was similar for two groups

**Group 1 results: **In group 1, peak cortisol response was <18 mcg/dL in 15 (16%) patients, 18-21 µg/dL in 19 (21%) patients and ≥22 mcg/dL in 53 (60%) patients (Table 1). Of the patients treated in group 1, nine had pituitary insufficiency and three had developed AI after cessation of steroid therapy.

**Figure 2:**
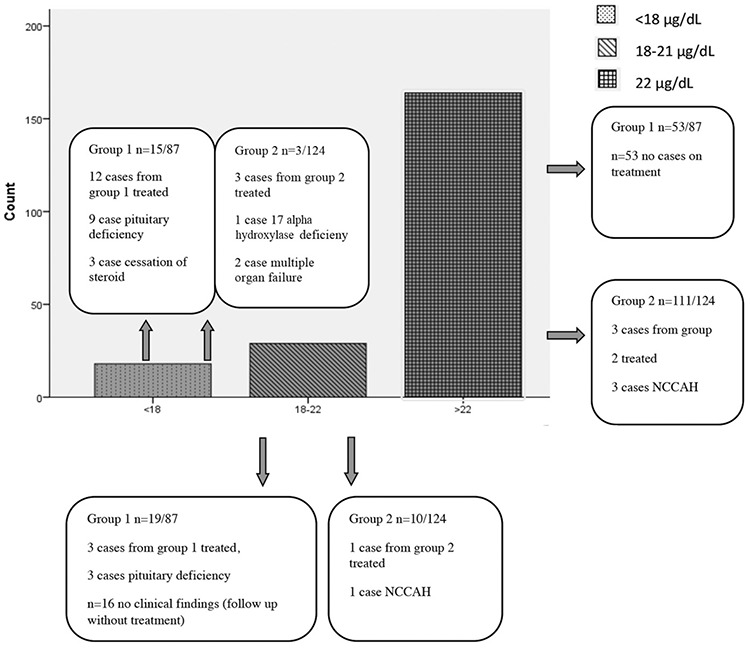
Number and treatment status of all cases according to peak cortisol level ITT: insuline tolerance test, NCCAH: non-classical congenital adrenal hyperplasia

Three of the patients with a borderline peak cortisol of 18-21 µg/dL were diagnosed as multiple pituitary hormone deficiency (MPHD) and received treatment. Peak cortisol response was <19 µg/dL in a further three. In the remaining 16 patients, peak cortisol response was 19-<22 µg/dL and these patients were followed without treatment since there was no clinical finding suggesting cortisol replacement would be appropriate (see Figure 2).

**Group 2 results: **In group 2, peak cortisol response was <18 µg/dL in three (3%) patients, 18-21 µg/dL in 10 (8%) and ≥22 µg/dL in 111 (60%) patients (Table 1). Three of the patients treated in group 2 with peak cortisol <18 µg/dL were diagnosed with 17 hydroxylase deficiency and two patients had multiple organ deficiency.

Multiple organ dysfunction syndrome is altered organ function in an acutely ill patient requiring medical intervention to achieve homeostasis. One of the patients with a peak cortisol of 18-21 µg/dL was diagnosed as NCCAH. No patient was detected with a peak cortisol response above 19 mcg/dL who also exhibited clinical signs of AI. On follow up, no patient with a peak corisol of >19 mcg/dL after im-ACTH test required treatment.

A total of 24 patients with a peak cortisol <18 mcg/dL after ITT, were evaluated with im-ACTH test and four of 24 (16.7%) patients showed an inadequate cortisol response and treatment was started. Five patients had a peak cortisol response of 18-21 µg/dL but none of them had any clinical findings and/or risk factors for AI. Fifteen patients had a peak cortisol level ≥22 µg/dL. When the gold standard was taken as clinical diagnosis; when a peak cortisol <18 µg/dL was used to define AI the following results were obtained: sensitivity 93%; specificity 99%; PPV 93%; and NPV 99%. Similarly, when AI was defined as peak cortisol <22 µg/dL the same parameters were: sensitivity 100%; specificity 86%; PPV 40%; and NPV 100% (see Table 2).

On follow up, 15 patients needed cortisol therapy whose peak cortisol was <18 µg/dL. In the whole cohort 3.3% (n=8) had 17-OHP ≥10 ng/dL. NCCAH clinic findings and/or mutation were found in six of these cases. The clinical findings and the test results of the cases were consistent. Mutations were detected in the 21 hydroxylase gene in 6 of 8 (75%) patients who were diagnosed as NCCAH with 17-OHP >10 ng/mL in group 2 as follows: *V281L* mutation in four, Intron 2 mutation in two, and the nine most common mutations were not present in the remaining two. No local and systemic side effects or allergic reactions were observed in any patient undergoing im-ACTH test.

## Discussion

There are no studies comparing im and iv ACTH tests for the detection of AI in childhood. The aim of this study was to assess the effectiveness of the im-ACTH test due to difficulty in obtaining iv-ACTH preparations in Turkey.

Due to limited access to IV Synacthen**^®^** (Novartis**^®^** Pharma AG 250 mcg/mL) clinicians have to make the diagnosis of AI with basal cortisol and ACTH. Although suspected cases are evaluated with basal ACTH and morning cortisol, this does not always provide a definitive diagnosis. The sensitivity of basal cortisol for the diagnosis of AI is only 60% (2). There are a number of reasons for this. These include immaturity of the hypothalamo-pituitary-adrenal (HPA) axis, very young patients, innaccurate sampling times for the morning cortisol which, because of diurinal cortisol variation, should be performed at a consistent time and thus differences between studies can be difficult to reconcile.

There are some adult studies describing assessment of the HPA axis with im-ACTH due to difficulties in obtainig the iv form and the poor reliability of the diagnosis of AI based on basal cortisol values. The efficacy of im-ACTH has been evaluated in detecting both primary and central AI cases in India (3). Gundgurthi et al (3), performed STT tests with im-ACTH (25 U im preformed with Acton). Two groups were identified; the validation group which included healthy volunteers, diabetes mellitus, hypothyroidism and AI and the study group, which included all patients who were tested because of a suspicion of AI. When the basal cortisol level was less than 3 µg/dL, it was diagnostic, but in the study, only 40% of those diagnosed with AI had basal cortisol levels below 3 µg/dL. In seven cases with a baseline cortisol value <3 µg/dL, the delta, defined as the difference between basal and peak cortisol in the test, increased by 7 µg/dL on stimulation. In eight cases, there was less increase but basal cortisol level was found to be >3 µg/dL. Subnormal increases have a similar sensivity to basal cortisol and have lower specificity and PPV. The authors concluded that the diagnosis of AI with im-ACTH form was superior to basal cortisol values and the HPA axis could be assessed with these tests in many health institutions. In addition, it was suggested that the im-ACTH test may be preferred because it was cheap and easily accessible.

Among our cases, 11 cases with basal cortisol ≤3 µg/dL indicating suspicion of AI were found. In only six of these eleven cases, peak cortisol value was <18 µg/dL and only seven cases required treatment.

Although there are limited studies in children, there are studies investigating the effectiveness of im and iv forms of ACTH in adults. Kelestimur et al (4) evaluated twenty healthy adults who were tested with IV Synacthen**^®^** and two weeks later with Depot Synacthene R. Thus the authors evaluated peak cortisol acquisition times and cortisol increase rates in the same volunteers using both iv and im forms of ACTH. Samples were taken at 0, 30, 60, 90 and 120 minutes and the time to obtain cortisol increase and peak response were similar. They concluded that depot forms of ACTH for SST may successfully replace iv Synacthen**^®^**. In 90% of cases tested with iv Synacthen**^®^**, peak responses were obtained ≥60 minutes and with the im form peak responses were detected at ≤90 minutes. When the peak response was taken as 22 µg/dL, the peak response was obtained in 95% of the patients who received both the iv and im form at 90 minutes or before. The only difference was that peak responses were concentrated at 30 minutes with the iv form and at 60 minutes using the im form. Thus, regardless of the form of synacthen^®^ used, there was no need to prolong the test to 120 minutes or more. In our study, blood was taken at 0, 30 and 60 minutes with the im form and peak responses were reached at 60 minutes. In our study, sampling was stopped after the 60 minute sample. We did not take a blood sample after 60 minutes.

Data obtained from adult studies are similar. Women of reproductive age (n=29) were given ACTH, either iv or im, and cortisol and androgen precursors were tested and no difference was found between the stimulated peaks obtained with the two forms (5). In another study, using im and iv forms, cortisol increase was compared with serum levels every 15 minutes and peak cortisol levels were obtained for both iv and im-ACTH forms at 60 minutes and the two forms were reported to be no different (6).

There are also studies attempting to determine the lowest effective dose of im-ACTH for the stimulation of the HPA axis. In one study, 21 healthy volunteers, nine primary adrenal AI and ten secondary AI were given im-ACTH (250 µg/mL Synacthen**^®^** NovartisR Pharma AG) at a standard dose of 250 µg im into the deltoid muscle and subsequently blood, saliva cortisol and aldosterone concentrations were measured (7). Standard dose was used for the study because it was conducted in adults.

In this study the doses were varied and titrated so that 12.5, 25 and 250 µg doses were compared. In healthy humans, 30 minute cortisol response was the same after 25 and 250 µg im while 12.5 µg was not sufficient to detect AI. Neither did the 30 minute salivary cortisol concentration differ between the 25 and 250 µg im injections. However, there was no correlation between salivary and blood cortisol levels. The responses were similar with no change in serum and salivary cortisol levels after low (25 µg) and high dose (250 µg) im ACTH in patients with AI.

The effectiveness of the im-ACTH test in detecting central AI cases has also been investigated (8). Cases with short stature and at risk of having multiple hormone deficiencies and tested with ITT were evaluated. Twenty cases were identified because of ITT peak cortisol below <18 µg/dL. All cases who responded poorly to ITT were re-evaluated one week later with 25 U ACTH and cortisol responses in both tests were compared. Although the peak responses were similar, an adequate response was obtained by im ACTH in six cases although there was insufficient response by ITT. This was attributed to the injection of ACTH at supraphysiological doses. A further finding of this study was that when the cut-off value was taken as 18 mcg/dL, sensivity was 57% with a specificity of 94% whereas the sensitivity increases to 100% when the cut-off was 22 µg/dL although the specificity fell to 75%. Thus the diagnosis of AI with a peak cortisol <18 µg/dL by im ACTH was robust whereas high reliability was ruled out at peak values above 22 µg/dL. In our cohort, in 24 cases with ITT cortisol response <18 mcg/dL, when the im ACTH test was done subsequently, the peak response was between 18-21 µg/dL in five patients and above 22 mcg/dL in 15 of them (it means that five patients have 22 and above 22) Only four patients underwent hydrocortisone replacement. There are two possible explanations. Firstly, the dose used for im ACTH may be a supraphysiological dose for central AI detection. Secondly, there was no central AI in any of the patients with a peak cortisol level <18 mcg/dL by ITT (9). It is known that the ITT response is age-variable and threshold values are not standard for all ages. This is supported by the finding that higher peak values were obtained in healthy children under the age of 12 years (9). Our second hypothesis supports the lack of AI findings in any of our patients who had insufficient response with ITT and had adequate response with im ACTH test.

### Study Limitation

The most important limitation of our study was that retest with iv ACTH could not be performed to evaluate the correlation with the im form of ACTH in our patients.

## Conclusion

In conclusion we have shown that the im form of ACTH test in children with suspected AI is a safe, effective and reliable test. None of the patients with peak cortisol levels ≥22 µg/dL were found to have AI on long-term follow-up. In contrast, peak responses <18 µg/dL were diagnosed as AI. Thus a peak response above 22 µg/dL excluded the diagnosis of AI.

## Figures and Tables

**Table 1:**
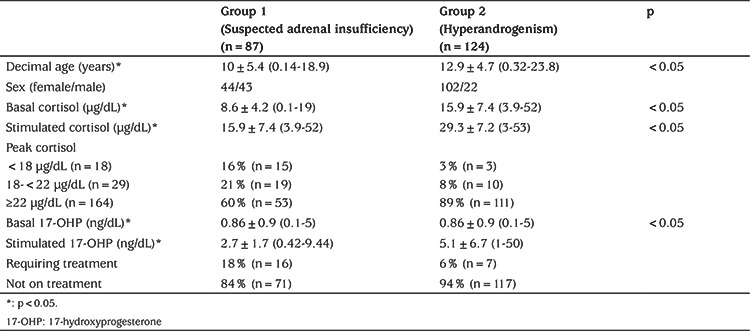
Demographic and biochemical characteristics of patients with intramuscular-adrenocorticotropic hormone test

**Table 2:**
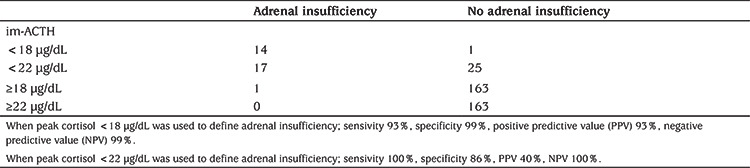
Threshold value after intramuscular-adrenocorticotropic hormone in patients with insulin tolerance test and peak cortisol <18 μg/dL
